# Evidence for multiple alleles effecting muscling and fatness at the *Ovine *GDF8 locus

**DOI:** 10.1186/1471-2156-8-80

**Published:** 2007-11-08

**Authors:** James W Kijas, Russell McCulloch, Janelle E Hocking Edwards, V Hutton Oddy, Sang Hong Lee, Julius van der Werf

**Affiliations:** 1 CSIRO Livestock Industries, Level 5 Queensland Bioscience Precinct, 306 Carmody Road, St. Lucia 4067, Australia; 2 South Australian Research and Development Institute, Struan Research Centre, Naracoorte, 5271, Australia; 3 School of Meat Science, University of New England, Armidale 2351, Australia; 4 School of Rural Science and Agriculture, University of New England, Armidale 2351, Australia

## Abstract

**Background:**

The current investigation surveyed genetic polymorphism at the ovine *GDF8 *locus and determined its contribution to variation in muscling and fatness in sheep.

**Results:**

Re-sequencing 2988 bp from a panel of 15 sires revealed a total of six SNP, none of which were located within exons of the gene. One of the identified SNP, *g+6723G>A*, is known to increase muscularity within the Belgian Texel. A genetic survey of 326 animals revealed that the mutation is near fixation within Australian Texels and present in additional breeds including White Suffolk, Poll Dorset and Lincoln. Using a resource population comprising 15 sires and 1191 half-sib progeny with genotypic data, the effect of this and other SNP was tested against a set of 50 traits describing growth, muscling, fatness, yield, meat and eating quality. The loss of function allele (*g+6723A*) showed significant effects on slaughter measurements of muscling and fatness. No effect was detected on objectively assessed meat quality however evidence was found for an association between *g+6723G>A*, decreased intramuscular fat and reduced eating quality. Haplotype analysis using flanking microsatellites was performed to search for evidence of currently unidentified mutations which might affect production traits. Four haplotypes were identified that do not carry *g+6723A *but which showed significant associations with muscling and fatness.

**Conclusion:**

The finding that *g+6723G>A *is present within Australian sheep facilitated an independent evaluation into its phenotypic consequence. Testing was conducted using a separate genetic background and animals raised in different environments to the Belgian Texel in which it was first identified. The observation that the direction and size of effects for *g+6723A *is approximately consistent represented a robust validation of the effects of the mutation. Based on observed allele frequencies within breeds, selection for *g+6723A *will have the largest impact within the White Suffolk. *GDF8 *may harbour additional mutations which serve to influence economically important traits in sheep.

## Background

Growth and differentiation factor 8 (*GDF8 *or myostatin) is known to directly influence muscular hypertrophy and carcass conformation. The gene is a member of the transforming growth factor-*β *superfamily of growth and differentiation factors and its role was first identified following gene targeting in the mouse [1]. The dramatic increase in both skeletal muscle fibre number (hyperplasia) and mass (hypertrophy) demonstrated *GDF8*'s role as a negative regulator of muscle growth. Naturally occurring *GDF8 *mutations were subsequently identified in two hyperplastic or 'double muscled' cattle breeds [2-4]. Investigation of additional double muscled cattle breeds identified allelic heterogeneity and a series of loss of function alleles [5].

Investigation of *GDF8*'s role in sheep has relied on association testing between linked microsatellite markers and variation in live-weight measures of muscling, fatness and carcass traits. Initial investigations using commercial New Zealand Texels reported high levels of marker homozygosity and effects on muscling and fatness [6]. Examination of half-sib progeny derived from British Texels revealed suggestive QTL affecting fat depth and muscling [7]. Analysis of a larger range of trait measurements in New Zealand Texels revealed that the strongest association was found for muscling and fatness traits in the leg [8]. This finding is consistent with a QTL segregating from the Belgian Texel investigated using F2 and backcross lambs created using Romanov ewes [9]. Analysis using this resource population culminated in the identification of a G to A transition in the 3' untranslated region of the gene which contributes to the muscular hypertrophy [10]. The presence of the mutant allele (*g+6723A*) creates an octamer recognition motif for the microRNAs, Mir1 and Mir206, which act to suppress *GDF8 *translation. This confirmed that removal of *GDF8*'s inhibitory role in sheep confers an increase in muscularity consistent with findings in other mammalian species.

Discovery of quantitative trait nucleotide (QTN) opens the possibility of using marker assisted selection to enhance genetic gain. As a result, the first aim of this study was to search for previously unidentified QTN at ovine *GDF8 *which contribute to economically important trait variation. The recent identification of *g+6723A *prompted testing to determine its frequency within 12 breeds and a set of Australian terminal sires. The final aim was to estimate the phenotypic effect of *g+6723A *on a range of carcass and meat quality traits measured under Australian conditions.

## Results

### Six SNP Identified by Re-sequencing

A set of 15 sires (Table 1) was used for re-sequencing to scan for loss of function mutations within the 1128 bp of translated sequence encoding the three *GDF8 *exons. No indels, synonymous or non-synonymous substitutions were detected, consistent with a single previous report for ovine *GDF8 *[11]. A further 1860 bp of the gene was investigated to search for putative regulatory mutations and SNP suitable for association analysis. Re-sequencing regions of the promoter (283 bp), intron 1 (480 bp), intron 2 (695 bp) and the 3' untranslated region (402 bp; UTR) revealed a total of 6 SNP. None appear to be located within splice sites which would interfere with transcriptional activity. Four have been identified previously and are located in the promoter (*g-41C>A*), intron 1 (*g+391G>T*), intron 2 (*g+4036A>C*) and the 3'UTR (*g+6723G>A*) [10]. The remaining two appear novel and are located within intron 1 (*g+2154C>T*) and the 3'UTR (*g+6871G>A*). The genotypic status, using IUB convention, at each polymorphism (ordered by numerical position) within the 15 sires (S1 – S15) was as follows: S1 CGCAGG, S2 CGCARG, S3 MGCAGG, S4 CGCAGG, S5 MGCAGG, S6 MGCAGG, S7 CGCAGG, S8 CGCAGG, S9 MGCAGG, S10 MKCMGG, S11 CGCARG, S12 MKCMGG, S13 MKCAGR, S14 CGCARG, S15 MKYMGG.

**Table 1:** Summary statistics for 15 half-sib families used to evaluate *GDF8*

			**GDF8 Haplotype **^3^					Hap
				
Sire	Breed ^1^	Progeny ^2^	1	2	3	4	5	ID ^4^
S1	P(24)	90	**186**	**C**	**A**	**G**	**139**	1
			204	C	A	G	135	2
S2	P(11)T(13)	95	**192**	**C**	**A**	**G**	**149**	3
			218	C	A	A	141	4
S3	P(15)	87	**186**	**A**	**A**	**G**	**141**	5
			216	C	A	G	151	6
S4	P(23)	80	**222**	**C**	**A**	**G**	**149**	7
			192	C	A	G	135	8
S5	P(28)	72	**204**	**C**	**A**	**G**	**135**	2
			188	A	A	G	149	9
S6	P(19)	92	**186**	**A**	**A**	**G**	**141**	5
			204	C	A	G	135	2
S7	P(18)	85	**186**	**C**	**A**	**G**	**139**	1
			192	C	A	G	149	3
S8	P(28)	43	**216**	**C**	**A**	**G**	**151**	6
			204	C	A	G	135	2
S9	W(28)	95	**184**	**C**	**A**	**G**	**139**	10
			188	A	A	G	151	11
S10	P(1)S(3)W(18)	53	**218**	**C**	**A**	**G**	**135**	12
			186	A	C	G	137	13
S11	P(1)S(5)T(3)W(21)	79	**218**	**C**	**A**	**A**	**141**	4
			218	C	A	G	137	14
S12	S(2)W(28)	81	**216**	**A**	**C**	**G**	**139**	15
			216	C	A	G	137	16
S13	P(1)S(9)W(10)	62	**192**	**C**	**A**	**G**	**135**	8
			214	A	A	G	149	17
S14	S(9)T(9)	95	**218**	**C**	**A**	**A**	**141**	4
			186	C	A	G	137	18
S15	P(19)	82	**212**	**C**	**A**	**G**	**149**	19
			216	A	C	G	143	20

^1 ^Male and female ancestors of each sire present within five generation pedigrees are given as P (Poll Dorset), S (Suffolk), T (Texel) or W (White Suffolk). The number of ancestors of each breed type is given in brackets.

^2 ^The number of half-sib progeny used for association analysis.

^3 ^*GDF8 *haplotypes for each sire comprise alleles at five markers encoded as follows: 1 (*BM81124*), 2 (*g-41C>A*), 3 (*g+4036A>C*), 4 (*g+6723G>A*) and 5 (*BULGE20*). Alleles are given in base pairs for the two microsatellite markers and nucleotides for the three SNP. Alleles which occur in phase are aligned horizontally and the two haplotypes in each sire are separated using a solid line.

^4 ^Haplotypes were numbered sequentially to allow comparison with haplotype effects (Table 6). Note that the loss of function mutation is present only within Hap4.

### Breed Distribution of *GDF8 *Alleles

Three SNP were selected for analysis within a larger set of animals (*g-41C>A*, *g+4036A>C *and *g+6723G>A*). The remaining SNP (*g+391G>T, g+2154C>T *and *g+6871G>A*) were not tested either due to low informativeness within sires 1 – 15 or unsuitability of assay design. Genotyping 326 animals from 12 breeds revealed that two SNP were polymorphic within every breed tested (*g-41C>A *and *g+4036A>C*, Table 2). The third SNP has been identified to cause muscle hypertrophy within Belgian Texel (*g+6723G>A*) [10]. Consistent with this finding, the loss of function allele was present in the homozygous form in 109 of the 116 Australian Texels tested (*g+6723A *p = 0.946, Table 2). This included sires sampled from 12 different producers and indicates the mutant allele is near fixation within commercial Australian Texel flocks. The allele was also present in breeds other than Texel. Testing of 77 White Suffolks, including 65 sires from 6 producers, revealed an allele frequency approaching 10% (p = 0.093, Table 2) while three Poll Dorset and one Lincoln were identified carrying *g+6723A *in the heterozygous form.

**Table 2:** Allele Frequencies for Three *GDF8 *SNP

Breed	N ^1^	*g-41A*	*g+4036C*	*g+6723A*
		freq	freq	freq
Black Suffolk	12	0.083	0.042	0.000
Coopworth	7	0.500	0.071	0.000
English Leicester	8	0.875	0.333	0.000
Lincoln	4	0.750	0.125	0.125
Merino	45	0.523	0.089	0.000
Poll Dorset	12	0.357	0.143	0.125
Polwarth	8	0.375	0.250	0.000
Romney	8	0.750	0.188	0.000
Southdown	8	0.500	0.188	0.000
Texel	116	0.028	0.009	0.946
Tibetan	21	0.475	0.024	0.000
White Suffolk	77	0.375	0.304	0.093
				
Totals	326	0.292	0.087	0.340

^1 ^Number of animals tested per breed.

^2 ^Frequencies are given for one of the alleles at each SNP (*g-41A*, *g+4036C *and *g+6723A*).

### *GDF8 *Haplotype Diversity

Two microsatellites expected to flank *GDF8 *(*BM81124 *and *BULGE20*) [8] and three SNP (*g-41C>A*, *g+4036A>C *and *g+6723G>A*) were used to assess haplotype diversity. Genotyping was performed using 15 Poll Dorset, White Suffolk or mixed breed sires (S1 – S15, Table 1) and their 1191 half-sib progeny. Both microsatellites were highly polymorphic with seven (*BULGE20*) and ten (*BM81124*) alleles observed within the 30 sire chromosomes tested. Marker segregation within family was used to assign allelic phase and construct five-marker haplotypes across the *GDF8 *locus (Table 1). A total of 20 different haplotypes were observed and none of the sires was homozygous indicating high levels of diversity. Table 1 shows that three sires were detected which carry the loss of function allele (*g+6723A*; sires S2, S11, S14). Inspection of the surrounding phase-known alleles at the other four markers revealed that in each case, *g+6723A *was observed on a single marker haplotype (*BM81124 *allele 218, *g-41C*, *g+4036A*, *BULGE20 *allele 141, Hap ID 4, Table 1).

### Breed of Origin of the Loss of Function Mutation

The breed history of each sire (S1 – S15) was determined from five generation pedigrees. Poll Dorset, Texel, Suffolk and White Suffolk ancestors were identified and the number of each is recorded in Table 1. Three of the 15 sires have a Texel ancestor (S2, S11 and S14). In each case this corresponded with the presence of *g+6723A*, suggesting Texel as the likely origin of the mutant allele. This is consistent with both the high frequency of *g+6723A *observed within Australian Texels (Table 2) and its identification in Belgian Texel [10]. Assuming Texel ancestry is a prerequisite for presence of the allele, Sheep Genetic Australia's phenotypic and pedigree database [12] was interrogated to determine the prevalence of Texel ancestors across nine major breeds. Five generation pedigrees were constructed for 5116 progeny tested sires and a total of 590 had at least one Texel ancestor (Table 3). Coopworth had the largest proportion of sires with Texel ancestors (69/130; 0.531) followed by White Suffolk (204/1853; 0.11) while three breeds (Border Leicester, Dorper and Merino) contained no recorded Texel relatives (Table 3).

**Table 3:** The proportion of Texel ancestors within Australian breeds

Breed	Sires ^1^	Sires with Texel ancestor ^2^	Prop. Sires Texel ancestor
Border Leicester	124	0	0.000
Coopworth	130	69	0.531
Poll Dorset	1942	11	0.006
Texel	291	291	1.000
Suffolk	264	4	0.015
White Suffolk	1853	204	0.110
Dorper	319	0	0.000
Merino	91	0	0.000
Commercial Terminals ^3^	102	11	0.108
			
Total	5116	590	0.115

^1 ^Number of sires with five generation pedigrees available for examination.

^2 ^Number of sires with at least one recorded Texel male or female ancestor.

^3 ^Composite or mixed breed sires used in the meat sheep industry.

### *GDF8 *SNP Association Tests Within the Resource Population

Eleven of the 15 resource sires were heterozygous for at least one of the three *GDF8 *SNP (*g-41C>A*, *g+4036A>C *and *g+6723G>A*, Table 1). This allowed linkage analysis to be performed using data collected on their half-sib progeny. Each SNP was genotyped across all 15 families (1191 progeny) before regression analysis was performed using 50 phenotypic traits (Table 4). Table 5 shows the majority of significant marker by trait associations were detected for *g+6723G>A*. The mutation had significant effects on muscling (P < 0.05 for loinwt, emac, emdc, emwc and forequarter) highly significant effects on fatness (P < 0.01 for cfatc and imf) and significant effects on subjective measures of eating quality (P < 0.05 for flavour, tender and satisfaction). The mutant allele (*g+6723A*) was associated with increased muscling, decreased fatness and a decrease in perceived eating quality (Table 5) but had no detected effect on shear force, ultrasonically derived muscling and fat depth, live weight or yield. Significant effects were detected for *g-41C>A *on forequarter weight (p < 0.05), meat tenderness (P < 0.05 for two shear measurements avkgf0 and diff072) and for *+4036A>C *on muscling (P < 0.05 for emwc).

**Table 4:** Summary of Phenotypic Traits

**Trait Description**	**Code**	**Mean**	**Unit**
Live Animal Traits			
Birth weight	bwt	5.9	kg
Pre-weaning weights	prewwt1	11.9	kg
	prewwt2	20.7	kg
Weaning weight	wwt	20.3	kg
Post weaning weights	pwwt1	30.3	kg
	pwwt2	29.6	kg
	pwwt3	30.9	kg
	pwwt4	33.6	kg
	pwwt5	39.4	kg
	pwwt6	45.1	kg
	pwwt7	52.8	kg
	pwwt8	49.1	kg
Final weight	flnwt	48.7	kg
Ultrasound fat depths	cfat1	2.6	mm
	cfat2	3.2	mm
Ultrasound muscle depths	emd1	27.2	mm
	emd2	28.4	mm
Carcass Traits			
Hot carcass weight	hcwt	21.9	kg
Eye muscle width	emwc	61.1	mm
Eye muscle depth	emdc	29.6	mm
Image traced eye muscle area	emac	16.0	cm^2^
Fat depth on carcass at C site	cfatc	3.3	mm
Fat and muscle depth at GR site	grknf	12.3	mm
Loin weight	loinwt	535.9	g
Viascan lean meat yield	lmy	11.6	kg
Yield of lean meat	yield	53.4	%
Weight of bone	bone	7.2	kg
Weight of trimmed fat	fat	4.2	kg
Loin fat weight	loinfatwt	374	g
Weight of boned forequarter	forequarter	3.4	kg
Weight of hind leg meat	leg	4.5	kg
Weight of tenderloin and shank	meat	3.1	kg
Objective Measures of Meat Quality			
Mean shear force at time zero	avkgf0	7.9	kgF
Mean shear force after 72 hours	avkgf72	3.2	kgF
Mean shear force after 5 days	avkgfsen	3.2	kgF
Difference in shear force at 0 and 72 hrs	diff072,	4.7	kgF
Total glycogen content at slaughter	gly	0.56	mg/100g tissue
Creatinine	creat	0.06	mg/dL blood
Total lactate present in the avkgf0 sample	lac	4.8	mg/g tissue
pH decline	phd	0.42	pH units/hr
Ultimate pH	phu	5.8	pH units
Lightness of the loin	l	35.6	units
Intramuscular fat content	imf	5.7	%
Sensory Measures of Eating Quality			
Liking of smell	likesmell	64.0	1 – 100
Tenderness	tender	59.6	1 – 100
Juiciness	juicy	52.0	1 – 100
Flavour	flavour	60.7	1 – 100
Hedonistic overall liking	ovlike	59.7	1 – 100
Satisfaction	satisfac	3.1	1 – 5
Composite overall score	seq	59.1	1 – 100

**Table 5:** Allele Contrasts for Three *GDF8 *SNP Significantly Associated with Phenotypic Traits

Trait	*GDF8 Allele *^1^	Effect ^2^	Effect in SD ^3^	Pr (> F-value)^4^
avkgf0 (k/F)	*g-41C*	0.459	0.232	0.021
cfatc (mm)	*g+6723A*	-0.509	-0.452	0.001
creat (mg/dL)	*g+6723A*	0.003	0.273	0.037
diff072 (k/F)	*g-41C*	-0.434	-0.225	0.025
emac (cm^2^)	*g+6723A*	0.589	0.329	0.015
emdc (mm)	*g+6723A*	0.706	0.267	0.045
emwc (mm)	*g+4036C*	-1.076	-0.369	0.038
emwc (mm)	*g+6723A*	1.077	0.369	0.006
forequarter (kg)	*g-41C*	-0.095	-0.416	0.024
forequarter (kg)	*g+6723A*	0.159	0.693	0.043
grknf (mm)	*g+6723A*	-1.161	-0.442	0.001
imf (%)	*g+6723A*	-0.631	-0.497	0.000
loinwt (g)	*g+6723A*	18.564	0.303	0.024
flavour	*g+6723A*	-2.943	-0.374	0.018
tender	*g+6723A*	-3.188	-0.354	0.025
satisfac	*g+6723A*	-0.109	-0.318	0.045

^1 ^The associated allele.

^2 ^Regression coefficient of the single marker allele on the trait.

^3 ^Proportion of phenotypic standard deviation.

^4 ^P-value for F-test.

### Estimation of Haplotype Effects Within the Resource Population

To search for evidence of function alleles other than *g+6723A*, microsatellites *BM81124 *and *BULGE20 *were genotyped across all 15 half-sib families. Haplotype effects were estimated for a set of five widely measured live-animal and carcass traits using both across-family and within-family analysis (Table 6). The microsatellite haplotype carrying *g+6723A *(Hap4, Table 1) showed significant across-family effects (Table 6) for the same set of traits identified by single marker analysis (Table 5). Comparison of Hap4 effects between sires (within-family analysis) revealed inconsistent effects for every trait. Six haplotypes which do not contain *g+6723A *showed significant effects. Two of these (Hap3 and 18, Table 6) were present in sires which carry Hap4 and their associated within-family effects arise from the indirect impact of *g+6723A*. The remaining four haplotypes, however, were identified in wildtype sires and suggest additional and currently unidentified functional alleles may be present at the ovine *GDF8 *locus. Two haplotypes showed effects on ultrasound eye muscle depth (emd1; Hap2 and Hap6), one on slaughter measurements of fat and muscle (grknf; Hap1) and one on eye muscle area (Hap13, Table 6). In nearly every case, within-family analysis revealed inconsistent associations between sires which carried the same haplotype (Table 6).

**Table 6:** Within and Across Family Estimation of Microsatellite Haplotype Effects

Hap ID ^1^	Sire	Trait ^2^	Across Family^3^		Within Family^4^	
				
			Effect	Pr (> LR-value)	Effect	Pr (> LR-value)
1	S1	grknf	-0.355	0.018	-0.382	0.084
1	S7		-0.363	0.017	-0.595	0.015
2	S1	emd1	0.280	0.028	0.280	0.202
2	S5		0.276	0.031	-0.010	1.000
2	S6		0.284	0.025	0.461	0.038
2	S8		0.283	0.028	0.244	0.438
3	S2	cfatc	0.339	0.039	0.574	0.008
3	S7		0.327	0.049	0.189	0.436
3	S2	grknf	0.555	0.001	0.564	0.010
3	S7		0.560	0.001	0.595	0.015
4	S2	emac	0.286	0.052	0.133	0.539
4	S11		0.292	0.052	-0.028	1.000
4	S14		0.302	0.041	0.620	0.005
4	S2	imf	-0.550	0.000	-0.414	0.059
4	S11		-0.564	0.000	-0.366	0.198
4	S14		-0.562	0.000	-0.778	0.001
4	S2	cfatc	-0.439	0.003	-0.574	0.008
4	S11		-0.446	0.003	-0.471	0.090
4	S14		-0.426	0.004	-0.171	0.437
4	S2	grknf	-0.427	0.004	-0.564	0.010
4	S11		-0.437	0.004	-0.606	0.026
4	S14		-0.417	0.005	-0.233	0.283
6	S3	emd1	-0.534	0.002	-0.711	0.001
6	S8		-0.530	0.003	-0.244	0.438
13	S10	emac	-0.351	0.029	-0.141	0.644
18	S14		-0.360	0.023	-0.620	0.005

^1 ^The genotypic composition of each haplotype is given in Table 1.

^2 ^Haplotype effects are given where significant likelihood ratio (LR < 3.84) was observed for any of five traits in either the across- or within-family analysis.

^3 ^Across-family haplotype effects were calculated using linkage and linkage disequilibrium (LDL).

^4 ^Within-family haplotype effects were calculated using linkage alone.

## Discussion

The current investigation focussed on genetic polymorphism at the *GDF8/myostatin *locus and its contribution to variation in muscling and fatness in sheep. The recent report of a loss of function *GDF8 *mutation (*g+6723A*) [10] prompted testing of 326 animals from twelve breeds to determine the frequency of *g+6723A *in Australian flocks. The allele appeared near fixation in a sample of 116 Australian Texels (Table 2), indicating that there is very little prospect for improving genetic gain in Texel through implementation of a diagnostic testing regime targeting g+6723A. The potential appears much greater in White Suffolk, where the allele frequency among 72 breeding sires was approximately 10%. Testing ten additional breeds revealed that the mutation was present within Poll Dorset and Lincoln, suggesting dispersal of the favourable allele has occurred across breed boundaries on multiple occasions. The breed of origin of the mutation remains unclear, however the finding that it occurs at high frequency in Australian Texel (Table 2) and was initially identified within Belgian Texel strongly suggests Texel sires have been responsible for transferring the allele into other breeds. To assess the likely dispersal across Australia's national flock, a pedigree analysis was performed to determine the proportion of sires which have at least one recorded Texel ancestor. Analysis of Coopworth showed over half have a known Texel relative (Table 3). Genotyping failed to detect the presence of *g+6723A *in Coopworth, however this was likely due to the small number of animals tested (n = 7, Table 2). Interestingly, the result for White Suffolk (204/1853 or 0.11, Table 3) closely matched the observed allele frequency (0.09, Table 2). Inspection of pedigree records for nearly 2000 Poll Dorset sires showed very few contained Texel ancestors (11). This suggests the frequency of the mutant allele is likely to be lower than that observed within the 12 animals genotyped for *g+6723G>A *(Table 2). It is possible, however, that unrecorded Texel ancestors exist or that breeds other than Texel have been responsible for introduction of the allele. A larger sample of Poll Dorset is required for genotypic assessment before a firm conclusion can be drawn.

A major objective of the study was to search for association between *GDF8 *alleles and variation in phenotypic traits. Regression analysis was performed using data collected from three SNP and 50 phenotypic traits measuring growth, muscling, fatness, meat quality and eating quality. Strikingly, segregation at *g+6723G>A *accounted for 12 of the 16 significant marker by trait associations detected (Table 5). No correction was made to account for multiple testing, however, the 12 significant *g+6723G>A *associations exceeds the expected number of false positive results (2.5) and gives an expected false discovery rate of only 20% [13].

The mutant allele (*g+6723A*) was associated with increased muscularity and decreased fatness measured on the carcass. It is worth noting that the half-sib progeny used were developed from Merino ewes and raised under Australian environmental conditions. Previous work using both F_2 _and backcross progeny was performed under European conditions using Romanov ewes [9,10]. Despite these differences, the observed direction and size of effect for *g+6723A *was approximately consistent for carcass traits common to both studies. This conclusion represents a robust and independent validation of *g+6723G>A *effect on sheep carcass traits. In addition to carcass traits, ultrasound derived measures of muscling and fat depth were recorded when progeny were approximately seven and nine months of age. Testing revealed none were significantly associated, indicating that the production level impact of *g+6723G>A *on muscling and fatness was restricted to heavier animals. This differs with reported QTL located near *Myostatin *in both New Zealand and UK Texels where significant effects were detected for ultrasound measures in some but not all sires [7,8]. Live weights were recorded at 13 times points between birth and slaughter to evaluate growth rate. This is of interest because investigation of GDF8 deficient Piedmontese cattle revealed an increase in growth rate and progeny of two UK Texel sires showed evidence for elevated weight at eight weeks [7,14]. Testing in the current study revealed no association, indicating *g+6723G>A *does not have a large impact on growth or live weight under Australian conditions. The final trait categories measured meat quality, tenderness and eating quality as it is critical to determine if favourable alleles which promote muscling have an associated negative impact on other traits. Four biochemical measures of meat quality (pH decline, ultimate pH, glycogen content at slaughter and levels of lactate) showed no association. In addition, three time point recordings of Warner-Bratzler shear force indicate that allelic variation at *g+6723G>A *imposed no significant effect on meat tenderness. This result is entirely consistent with a detailed examination of meat quality conducted using New Zealand Texels segregating divergent haplotypes at *GDF8 *[15]. Interestingly, *g+6723A *was associated with elevated creatinine concentration which has been used as an indicator of total muscle mass and is elevated in sheep which carry a separate hypertrophy phenotype (*Callipyge*) [16]. Testing in the current study identified a significant negative effect for *g+6723A *on the percentage of intramuscular fat (Table 5). This has not been measured in previous studies [7-9,15]. Reduced intramuscular fat might be expected to impact on eating quality, and a significant effect was detected for three sensory measurements (Table 5). In each case, *g+6723A *was associated with a decrease in eating quality which raises the possibility that the increased muscularity may be accompanied by a lowered consumer preference. The effect of *g+6723A*, however, was not reflected in elevated shear force or any of the meat quality traits evaluated in this study. Together, these and previous results indicate marker assisted selection for an increased frequency of *g+6723G>A *should improve desirable traits in the carcass without compromising objective measures of meat quality.

The second major objective of the study was to search for evidence of additional QTN (other than *g+6723G>A*) with an impact on the traits of interest. This was addressed using two approaches. Firstly, each protein coding exon and splice junction were re-sequenced using DNA from each of the 15 sires which comprise the resource population. This revealed a complete absence of sequence differences and indicates that the Poll Dorset, Suffolk and White Suffolk are unlikely to carry exonic mutations at or near fixation. Investigation of sires from additional breeds is required before a firm conclusion can be drawn, however this appears to distinguish the ovine homolog of *GDF8 *from its bovine equivalent where a series of loss of functions alleles have been reported within the three coding exons [5]. The second approach utilized regression analysis using haplotypes derived from alleles at microsatellites *BM81124 *and *BULGE20*. The intention was to exploit the superior polymorphic information content resident at the microsatellite markers to search for haplotypes in phase with currently unidentified mutations. Two analytical methods were used. Firstly, haplotype effects in different families were correlated as the covariance was determined by haplotype similarity (across-family analysis, Table 6). The second method considered only linkage information and estimated haplotype effects independently within each half-sib family (within-family analysis, Table 6). The later approach is less accurate but also less restrictive, as QTL effects vary between families. As expected, the single haplotype which contained *g+6723A *(*BM81124 *allele 218 - *BULGE20 *allele 141, Hap4, Table 1) gave significance effects for multiple traits, even though segregation at *g+6723G>A *was not considered in the analysis (Table 6). The observation that its effects were inconsistent between sires has been previously observed [7,8,10]. Of particular interest were those associations involving haplotypes which do not carry *g+6723A *(Hap1-3, Hap6, Hap 13 and Hap 18, Table 6). It is important to note that Hap3 and Hap18 are present in sires which also carry Hap4 (sires S2 and S14, Table 1). The significant effects observed following within-family analysis therefore arise from the contrast with Hap4 and the action of *g+6723A*. This was not the case for the other haplotypes which were found to affect ultrasound measures of muscling and other carcass traits. Animals which carry these haplotypes are targets for expanded re-sequencing to search for regulatory mutations. Alternatively, the observation that additional *GDF8 *haplotypes have an effect on phenotype may indicate the involvement of tightly linked neighbouring genes consistent with the identification of a second QTL in the region [8].

## Conclusion

In order to conclude that an observed sequence variant is a QTN, a critical mass of evidence is required from linkage analysis, positional cloning, concordance analysis, functional studies and transgenics. The identification of *g+6723G>A *in sheep is one of the few putative QTN in livestock which appears to meet the required burden of proof [24]. True QTN are expected to maintain their effect when present within different genetic backgrounds [24]. This was tested in the current study, and the observation that the effect of allele *g+6723A *was maintained within mixed breed sires (S2, S11 and S14, Table 1) adds to the burden of proof supporting *g+6723G>A *as the QTN underlying the muscling QTL located on sheep chromosome 2 [6-8].

## Methods

### Sequence analysis of ovine GDF8

Six primer sets were designed from available ovine sequences [GenBank: AY918121, AF393618, AF266758] to span the three exons of the sheep *GDF8 *gene (primers MSTN1 - 6, Table 7). A seventh primer pair was designed from available cattle sequence (AF320998) to amplify a portion of the 3' untranslated region (MSTN7F/R, Table 7). Primer sets were used to amplify and sequence fragments from 15 sires (S1 – S15, Table 1). PCR was conducted in 20 *μ*l reactions containing 50 ng of template DNA, 0.5 *μ*M of both forward and reverse primer, 1.5 mM MgCl_2_, 200 *μ*M dNTP, 0.5 U HotStarTaq™ DNA Polymerase and reaction buffer (Qiagen, Victoria, Australia). Amplified products were directly sequenced in both forward and reverse orientation using ABI Prism™ Big Dye Terminator 3.2 chemistry and the ABI 3130xl Genetic Analyser (Applied Biosystems, Victoria, Australia). Sequence was trimmed and aligned using Sequencher version 4.2 (Gene Codes Corporation, Ann Arbor, MI, USA). SNP were named in relation to the first base in the ATG translation start site, as given in GenBank accession DQ530260[10].

**Table 7:** Primers used for *GDF8 *exon scanning and mutation detection

Oligonucleotide	Region	Sequence (5' – 3')
MSTN1-F	promoter-exon1	TGAATCAGCTCACCCTTGACT
MSTN1-R	promoter-exon1	GCTGCTGTCATCTCTCTGGA
MSTN2-F	exon 1 – intron 1	GCCATAAAAATCCAAATCCTCA
MSTN2-R	exon 1 – intron 1	ACAAGCCAGCAGCTTGTTAAA
MSTN3-F	intron 1 – exon 2	TTGTCCCCAGGTAATTCAGG
MSTN3-R	intron 1 – exon 2	GCCTGGGTTCATGTCAAGTT
MSTN4-F	exon 2 – intron 2	AGTAAAGGCCCAACTGTGGA
MSTN4-R	exon 2 – intron 2	TTGGGTACAGGGCTACCATC
MSTN5-F	intron 2 – exon 3	TGGAGTTCGTCTTTCCAACC
MSTN5-R	intron 2 – exon 3	CCCATCCAAAAGCTTCAAAA
MSTN6-F	exon 3 - 3' UTR	TTGGGCTTGATTGTGATGAG
MSTN6-R	exon 3 - 3' UTR	CTTAAGTGACTGTAGCATACTC
MSTN7-F	3' UTR	AGTGGTCTTAAAACTCC
MSTN7-R	3' UTR	CAATTTATTTGATTAACAAAATCCTGA
g-41C>A-F	*g-41C>A*	GTTTGGCTTGGCGTTACTCAA
g-41C>A-R	*g-41C>A*	CAAAGATTTGCAGTTTTTGCATGGTTTT
g-41A probe	*g-41C>A*	VIC-TTGCTCTTACTTCTTCC
g-41C probe	*g-41C>A*	FAM-TGCTCTTACTGCTTCC
g+4036A>C-F	*g+4036A>C*	GCAGTACTGGGTGGCAACTATTTTA
g+4036A>C-R	*g+4036A>C*	ATTCTCCATTCACTTGGTCTTGTTCA
g+4036A probe	*g+4036A>C*	VIC-TTAATATCCAAATTTCCC
g+4036C probe	*g+4036A>C*	FAM-ATCCACATTTCCC
g+6723G>A-F	*g+6723G>A*	GGTTCGTGATGGCTGTATAATGTGA
g+6723G>A-R	*g+6723G>A*	GATTTCAGATAATAGAGTTAAATCATTTTGGTTTGCTT
g+6723G probe	*g+6723G>A*	VIC-TTCAAATCTCAACGTTCCAT
g+6723A probe	*g+6723G>A*	FAM-TCAAATCTCAACATTCCAT
BM81124-F	microsatellite	GCTGTAAGAATCTTCATTAAGCACT
BM81124-R	microsatellite	CCTGATACATGCTAAGGTTAAAAAC
Bulge20-F	microsatellite	CAGCAGGTCTGTTGAAGTGTATCAG
Bulge20-R	microsatellite	AGTGGTAGCATTCACAGGTAGCCAG

### Analysis of *GDF8 *polymorphism

SNP detection was performed using the fluorogenic 5' nuclease assay formatted for analysis using Applied Biosystems (ABI) 7900HT 'TaqMan' sequence detection system (primer and probe sequences are recorded in Table 7 using prefix 'g'). Reactions were conducted in 10 *μ*l containing 2 ng of genomic DNA, 2.25 *μ*M of each PCR primer, 0.8 pM of each TaqMan probe and 5 *μ*l of TaqMan Universal Master Mix (ABI). Cycling was performed as follows: 2 min at 50°C and 10 min at 95°C; 45 cycles of 95°C for 30 sec and 60°C for 60 sec. End point allele discrimination analysis was performed using the Sequence Detection System (SDS) software version 2.2.2 (ABI). Primers for amplification of microsatellite markers *BM81124 *and *BULGE20 *(Table 7) were synthesized with fluorescent tags NED and VIC respectively. PCR was performed as described for exon re-sequencing before fragment sizes were determined using the ABI 3130XL and associated GeneMapper software version 3.7 (Applied Biosytems).

### Breed Distribution of *GDF8 *Alleles

Allele frequencies at three *GDF8 *SNP were determined within each of 12 breeds (Table 2). The DNA samples used have been described previously [17]. Additional population samples from the Australian Texel (n = 111) and White Suffolk breeds (n = 65) were collected from commercial sheep farmers as part of this work. Blood was collected on Guthrie cards before DNA extraction and whole genome amplification was performed using the 'Genomiphi' DNA kit according to the manufacturers instructions (Amersham Biosciences). Genotyping of all animals was performed using 'TaqMan' SNP assays described above.

### Analysis of Sire Pedigrees

Pedigree records from 5116 progeny tested sires were obtained from Sheep Genetics Australia (SGA) which provides a national genetic information and evaluation service to the sheep industry [12]. Each pedigree was searched for the presence of Texel ancestors (Table 3).

### Resource Population and Phenotypic Trait Measurement

Fifteen half sib families were generated using Poll Dorset, White Suffolk or mixed breed sires (the breed composition of each sire is presented in Table 1). The sires were drawn from Australian industry flocks and mated with Merino ewes. A total of 1566 progeny were born in 2000 and 2001 at the Struan Research Center, South Australia. The description, code, trait mean and units of measurement for each trait are given in Table 4. Live weight was recorded at birth (bwt) and at approximately 30 day intervals (prewwt1, prewwt2, pwwt1 - 8) until a final weight was recorded at day 300 (flnwt). Ultrasound measurements of fat and eye muscle depth were recorded at approximately day 210 (cfat1, emd1) and day 260 (cfat2, emd2). A total of 1224 progeny were slaughtered at approximately 300 days of age and measured for hot carcass weight, eye muscle width, eye muscle depth, eye muscle area, fat depth at the C site, loin weight, lean meat yield as measured by Viascan and fat and muscle depth at the GR site. A subset of carcasses (350) was dissected into their component parts in a boning room to generate the yield of lean meat, weight of bone, weight of trimmed fat, loin fat weight, weight of boned forequarter, weight of hind leg and weight of remaining meat including tenderloin and shank. Objective measurements of eating quality included mean Warner Bratzler shear force at zero, 72 hrs and 5 days post slaughter [18], total glycogen at slaughter [19], total lactate, pH decline, ultimate pH, lightness of the loin measured by chromameter and intramuscular fat content predicted by near infrared spectrophotometry following calibration for sheep [20]. Sensory measurements of subjective eating quality traits were collected on samples from 528 progeny using methods described elsewhere [21]. Ten consumers rated liking of smell, tenderness, juiciness, flavour and hedonic overall liking using a range of 1–100. Satisfaction was ranked using a range of 1 – 5. For each trait, higher scores indicate favourable sensory perception and the averaged score from all ten consumers was used. A composite score was calculated which comprised 0.2.Tender + 0.1.Juicy + 0.3.Flavour + 0.4.Ovlike.

### Statistical Analysis

Regression analysis of phenotypes on single marker allele variants was performed, fitting three SNP simultaneously (*g-41C>A*, *g+4036A>C *and *g+6723G>A*, Table 5). The model was:

yi=μ+si+∑k=1nmarker⁡sbkgijk+ei
 MathType@MTEF@5@5@+=feaafiart1ev1aaatCvAUfKttLearuWrP9MDH5MBPbIqV92AaeXatLxBI9gBaebbnrfifHhDYfgasaacPC6xNi=xI8qiVKYPFjYdHaVhbbf9v8qqaqFr0xc9vqFj0dXdbba91qpepeI8k8fiI+fsY=rqGqVepae9pg0db9vqaiVgFr0xfr=xfr=xc9adbaqaaeGacaGaaiaabeqaaeqabiWaaaGcbaGaemyEaK3aaSbaaSqaaiabdMgaPbqabaGccqGH9aqpiiGacqWF8oqBcqGHRaWkcqWGZbWCdaWgaaWcbaGaemyAaKgabeaakiabgUcaRmaaqahabaGaemOyai2aaSbaaSqaaiabdUgaRbqabaGccqWGNbWzdaWgaaWcbaGaemyAaKMaemOAaOMaem4AaSgabeaakiabgUcaRiabdwgaLnaaBaaaleaacqWGPbqAaeqaaaqaaiabdUgaRjabg2da9iabigdaXaqaaiabd6gaUjabd2gaTjabdggaHjabdkhaYjGbcUgaRjabcwgaLjabckhaYjabdohaZbqdcqGHris5aaaa@538F@

where y_ij _is the phenotype on animal j from sire i, s_i _is the polygenic effect of the sire and g_ijk _is the paternal allele code (0 or 1) for the marker allele that animal j received from sire i for the k^th ^marker, and b is the regression coefficient (estimated marker effect) of marker k. Phenotypes were pre-adjusted for relevant fixed effects (birth year, management group, birth type, and sex) using an additive linear model. Slaughter traits were also corrected for effect of batch (9 slaughter batches over 2 years) and eating quality traits were corrected for test night (15 levels), birth-year and sex. Marker regression analysis was performed using the R-Program [22]. In subsequent analysis, two models were used to determine the association between phenotypes and haplotypes constructed using variation at microsatellites *BM81124 *and *BULGE20*. In both models, regression was used where each sire haplotype was fitted under the hypothesis it carried a QTN. Identity by Descent (IBD) probabilities were calculated for each putative QTL at the midpoint between *BM81124 *and *BULGE20*. In the first model, haplotype contrasts were calculated independently within each of the 15 half-sib families. The covariance between identical haplotypes in different families was set to zero, implying that the QTL effects were allowed to vary between sire families. The resulting solutions for haplotype effects are referred to as the 'within-family' analysis (Table 6). In the second model, haplotype effects across families were correlated, with the covariance determined by haplotype similarity using previously published methods [23] ('across-family analysis', Table 6). For both models, a likelihood ratio (LR) test was calculated, indicating the ratio of log likelihoods of a model with haplotype effect over a model without haplotype effects.

## Authors' contributions

JWK conceived and guided the study, carried out the exon scanning and drafted the manuscript. RM performed DNA extractions the genotyping of all the animals. JEHE coordinated construction of the half-sib resource flocks and collection of phenotypic trait data. VHO participated in study design, data coordination and manuscript preparation. SHL carried out the association and haplotype analysis. JVDW contributed to study design, manuscript preparation and coordination of the statistical components of the work. All authors read and approved the manuscript.
